# Generation and the subjective feeling of “aha!” are independently related to learning from insight

**DOI:** 10.1007/s00426-015-0697-2

**Published:** 2015-08-18

**Authors:** Jasmin M. Kizilirmak, Joana Galvao Gomes da Silva, Fatma Imamoglu, Alan Richardson-Klavehn

**Affiliations:** 1 Memory and Consciousness Research Group, Department of Neurology, Otto-von-Guericke-University Magdeburg, Leipziger Str. 44 (Haus 65), 39120 Magdeburg, Germany; 2 CogNovo, Plymouth University, Devon, UK; 3 International Computer Science Institute, Berkeley, CA USA; 4 Helen Wills Neuroscience Institute, University of California, Berkeley, Berkeley, CA USA; 5 Berkeley Institute for Data Science, University of California, Berkeley, Berkeley, CA USA

## Abstract

It has been proposed that sudden insight into the solutions of problems can enhance long-term memory for those solutions. However, the nature of insight has been operationalized differently across studies. Here, we examined two main aspects of insight problem-solving—the generation of a solution and the subjective “aha!” experience—and experimentally evaluated their respective relationships to long-term memory formation (encoding). Our results suggest that generation (generated solution vs. presented solution) and the “aha!” experience (“aha!” vs. no “aha!”) are independently related to learning from insight, as well as to the emotional response towards understanding the solution during encoding. Moreover, we analyzed the relationship between generation and the “aha!” experience and two different kinds of later memory tests, direct (intentional) and indirect (incidental). Here, we found that the generation effect was larger for indirect testing, reflecting more automatic retrieval processes, while the relationship with the occurrence of an “aha!” experience was somewhat larger for direct testing. Our results suggest that both the generation of a solution and the subjective experience of “aha!” indicate processes that benefit long-term memory formation, though differently. This beneficial effect is possibly due to the intrinsic reward associated with sudden comprehension and the detection of schema-consistency, i.e., that novel information can be easily integrated into existing knowledge.

## Introduction

Solving problems by insight has been proposed to be beneficial to long-term memory (LTM) encoding (Auble, Franks, & Soraci, 1979; Danek, Fraps, von Müller, Grothe, & Ollinger, 2013; Ludmer, Dudai, & Rubin, 2011), a proposal that could have considerable educational as well as neuroscientific relevance. It would suggest that educators can optimize learning by actively creating situations during which their students experience insight. Understanding the relevant cognitive processes could help devising such educational strategies, in addition to addressing the fundamental brain basis of learning and memory. However, insight has been operationalized very differently across studies (e.g., Auble et al., 1979; Bowden, Jung-Beeman, Fleck, & Kounios, 2005; Kounios & Beeman, 2009; Ludmer et al., 2011). For example, Mednick operationalized insight as solving a problem that requires creativity (Mednick, 1962), whereby creativity was understood as the process of searching successfully for a unique common associate between a provided set of three words that are only remotely associated by that word (e.g., cottage, blue, goat–cheese). If the words were already closely associated, it would not require creativity to find the missing link. Others have asserted that the presence of a subjective “aha!” experience is necessary to interpret a solution to a problem as an insight (Bowden & Jung-Beeman, 2003a; Bowden et al., 2005; Danek et al., 2013). In contrast to this definition, Mednick’s operationalization is relatively more objective as the correctness of a solution to a problem that has only one possible solution can be judged by a third person. Again others required only a state of previous incomprehension of the solution to a problem, followed by a sudden change to a state of understanding the solution, which could even be induced by presenting the solution (Auble et al., 1979; Ludmer et al., 2011). Finally, some researchers have proposed that insight does not necessarily include a state of incomprehension, but needs mental restructuring (Wills, Estow, Soraci, & Garcia, 2006), for which the common term in the insight literature is a representational change of the problem/solution space (Ohlsson, 1984; Öllinger, Jones, & Knoblich, 2013). Therefore, it is difficult to say by what means insight problem-solving may facilitate encoding.

In the current study, we are focusing on two aspects of the different operationalizations of insight that may be especially relevant to learning, but which are not used consistently: generation, that is, whether the solution to an insight problem is found by the participant or presented by the experimenter, and the subjective “aha!” experience,^1^ which is a subjective feeling of comprehending the solution suddenly while being convinced of its truth. We now address these components in turn and explain their relevance to learning.

### The generation effect

The *generation effect* is the superiority of items that are generated by participants over items that are presented to them in regard to later LTM performance (Burns, 1992; Slamecka & Fevreiski, 1983; Slamecka & Graf, 1978). Generation may involve, for example, coming up with a word associated to a cue word, completing a word stem, solving an anagram, finding an antonym, or—in terms of the question at hand—finding a solution to a problem in contrast to being presented with the solution. It has been consistently found that requiring generation, in comparison to presenting the solution, leads to enhanced later long-term memory as tested by recognition memory, cued recall, and free recall (e.g., Burns, 1992; McNamara & Healy, 2000; Slamecka & Graf, 1978). In addition, this effect is larger the longer the retention interval (for a summary and overview, see the meta-analysis by Bertsch, Pesta, Wiscott, & McDaniel, 2007). There have been several attempts to explain the effect, the main hypotheses being (1) cognitive effort, (2) transfer-appropriate processing, and (3) level of processing (LOP).

The first explanation suggests higher cognitive effort used to engage with the items in contrast to mere reading/perceptual processing. However, in an extensive meta-analysis, Bertsch et al. found no dependency on cognitive effort, that is, task difficulty (Bertsch et al., 2007). The second explanation, transfer-appropriate processing, refers to the finding that memory performance is better, the closer the processing required by the memory test corresponds to the processing that occurred in the encoding phase. However, the evidence shows only partial support for this idea (Bertsch et al., 2007). The third explanation, level of processing, is probably the strongest so far, that is, that generating an item leads to a more elaborate semantic processing of the item than reading the solution, thereby enhancing memory (McElroy & Slamecka, 1982; Slamecka & Graf, 1978). There is strong evidence that better semantic integration for the generate condition is at the core of the generation effect. When comparing words with non-words in a generate and read condition, there is no generation effect for non-words—not even when the generation rule is rhyming, that is, lexical-phonologic, or when the target word to a non-word can be a word (McElroy & Slamecka, 1982). This result suggests that the semantic spreading activation of related concepts during generation and a better integration of the generated items into existing semantic networks is mostly responsible for the later memory advantage (Anderson, 1983; Collins & Loftus, 1975).

### The subjective “aha!” experience

The “aha!” experience is defined as the sudden, unexpected comprehension of a solution to a problem that comes with an ease of understanding the solution. It is usually accompanied by a positive feeling that is not the feeling of pride to have solved the item, because it comes before the assessment of the solution. Moreover, there is complete confidence in the truth of the solution (Topolinski & Reber, 2010). The generation of a solution per se does not necessarily provide any information about how participants arrived at the solution, e.g., whether they arrived at it via sudden insight, by means of a analytical problem-solving approach, or otherwise—only if one assumes that a problem is not solvable without insight (such as Mednick, 1962). We therefore investigated not only the relationship between generating solutions to insight problems, but also how generation relates to the subjective “aha!” experience, and their possible interaction in regard to learning. Surprisingly, none of the authors who have previously used the subjectively reported “aha!” as a criterion for insight has reported whether such “aha!” experiences may also be reported when the solution is presented after a failed attempt at problem-solving.

The “aha!” experience was coined by Bühler (1907) who referred to it as a sudden verbal-cognitive understanding of a foreign idea/sentence. Something novel is learned by means of a complex interaction between one’s declarative memory content and problem-solving processes tackling the sentence. A more recent operationalization of the subjective “aha!” experience was introduced by Bowden and Jung-Beeman (2003b). Before the experiment, participants were provided with the following definition of an “aha!” experience: “A feeling of insight is a kind of ‘Aha!’ characterized by suddenness and obviousness. You may not be sure how you came up with the answer, but are relatively confident that it is correct without having to mentally check it. It is as though the answer came into mind all at once—when you first thought of the word, you simply knew it was the answer. This feeling does not have to be overwhelming, but should resemble what was just described”. (p. 507, Jung-Beeman et al., 2004). During the experiment, whenever the participants solved a problem, they were asked whether they solved it with an “aha!” experience or not. The authors interpreted items being solved without an “aha!” experience as being solved analytically, that is, by means of applying known problem-solving strategies and continuously getting closer to the solution until it has been found.

However, whether such a clear-cut binary categorization can be made is questionable. For example, Klein and Jarosz (2011) investigated naturally occurring insights, by collecting reports of such incidents in everyday life. They observed that reaching an impasse occurred only in some cases, although a change in understanding happened most of the time and helped coming up with new ways to approach solving the problem (representational change of the problem/solution space). Moreover, insights were often reached gradually: an intuitive feeling of coming closer to the solution often preceded the insight for a long time. During this time, inconsistencies with one’s own assumptions and with information that at first seemed coincidental was gradually perceived to form a pattern that finally led to the solution (Klein & Jarosz, 2011). They therefore proposed that the subjective “aha!” experience might be an epiphenomenon that accompanies some of the insight solutions, probably those that are found after an impasse has been suddenly overcome, but not all. On the other hand, Bowers suggests that the intuitive feeling of getting closer to the solution is due to automatic spreading activation from all known pieces of information to associated semantic information that gradually form coherence which at some point emerges into consciousness (Bowers, Regehr, Balthazard, & Parker, 1990). This theory of insight problem-solving does not necessitate impasse for the “aha!” experience which merely indicates the sudden emergence into consciousness of a hypothesis about the solution.

In the current study, we used a definition of the subjective feeling of “aha!” that was based on the Bowden and Jung-Beeman (2003a, b) definition, but changed it so that the generation of a solution was not declared a necessary precondition for “aha!”. This way, subjects were unbiased, therefore enabling us to test whether “aha!” experiences do indeed depend on generation. It should be noted that we did not specify the occurrence of an impasse or representational change as a defining criterion for the subjective “aha!” experience, because it is not clear whether it is necessary precondition as discussed above. Moreover, we do not see no “aha!” as analytical problem-solving but leave it undefined, as it is only clear that subjects did not comprehend the solution in the specified way for “aha!”, but not by what means they reached an understanding.

### Insight and long-term memory encoding

We assume that insight can enhance LTM encoding (Auble et al., 1979; Danek et al., 2013). There are several factors that may lead to such a positive influence on memory formation. First, generation can enhance LTM encoding for an item although, as previously discussed, the explanation for this effect is not completely understood. Thus, learning from insight may occur mainly due to the generation effect. Second, Auble et al., found that learning only occurs when an initial state of incomprehension is followed by comprehension (Auble et al., 1979). In that sense, we hypothesize that the subjective feeling of “aha!”, indicating the sudden change from incomprehension to comprehension, may also occur when the solution is presented after an unsuccessful attempt at problem-solving. However, an “aha!” experience during generation has been found to enhance encoding even more than generation without “aha!” (Danek et al., 2013). Third, the novelty of the sudden coherence of the solution may be another factor facilitating learning. Novelty responses in the brain are similar to reward responses; novelty itself can be seen as rewarding as summarized in the novelty exploration bonus hypothesis (Kakade & Dayan, 2002). Associative novelty and reward are both thought to facilitate memory encoding by means of increased dopaminergic input from the midbrain to the hippocampus (amongst other regions), which is a key region of memory consolidation (e.g., Bunzeck, Doeller, Dolan, & Düzel, 2012; Kumaran & Maguire, 2009; Lisman & Grace, 2005; Otmakhova, Duzel, Deutch, & Lisman, 2013). Thus, learning from insight may further be based on the rewarding feeling that is based on the suddenness of understanding a novel relationship that previously seemed incomprehensible and is accompanied by a positive emotional response. Lastly, evidence suggests that detecting novel information that fits into an existing schema can accelerate learning (Tse et al., 2007; Van Kesteren, Ruiter, Fernández, & Henson, 2012). This was in fact one of the original Gestalt theories on learning from insight, i.e., that learning from insight occurs as novel information builds a novel schema with information already present in semantic memory. The previously perceived ambiguity is suddenly resolved as the association becomes clear (Bühler, 1907; Vollmers, 2014). To use the example of Archimedes: he suddenly realized the connection between the water level in his bath tub rising and the mass submerged into the water, although all necessary pieces of information were already known to him.

The subjective “aha!” experience may reflect a specific quality of comprehension. It is possible that the subjective “aha!” experience is evoked by a particularly strong novelty response due to the suddenness of comprehension. Moreover, while successfully generating a solution may already evoke a positive emotional response, the “aha!” experience is additionally associated with positive affect (Danek et al., 2013; Topolinski & Reber, 2010).^2^ This positive emotional response may reflect the joy of comprehension or relief from the state of tension due incomprehension (Danek, Fraps, von Müller, Grothe, & Öllinger, 2014). We would therefore expect that the “aha!” experience per se can enhance memory formation, even when the solution is presented after failing to generate it, and when the additional positive emotional response related to solving the problem is therefore absent.

### Aims of the current study

In the current study, we investigated the extent to which the generation of a solution and the subjective “aha!” experience are associated with LTM encoding in regard to two different measures of memory. We further wanted to evaluate the qualitative difference of the sudden comprehension for generated and non-generated solutions, as well as solutions understood with an “aha!” experience and without. The latter was especially interesting, because most previous studies reflect the indirect assumption that the subjective “aha!” experience can only occur when the solution is generated instead of being presented, without looking at generation and “aha!” in a fully crossed design.

**Fig. 1:**
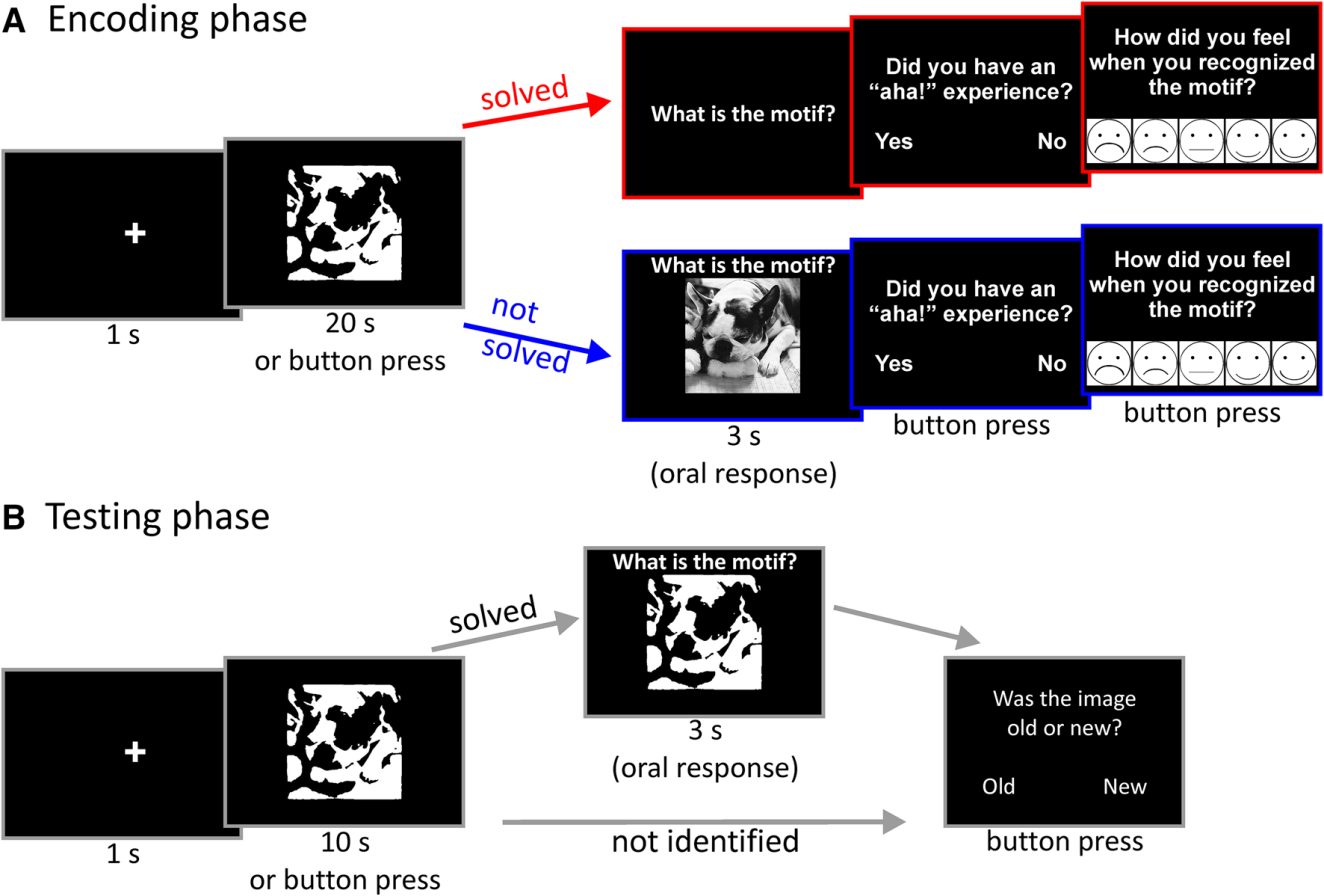
**a** *Encoding phase* In our incidental encoding task, participants were instructed to try to identify the motifs of the images. If identified, the motif should be named orally. If not identified, the original *grayscale* image was presented and participants should name the motif to avoid memory differences due to the oral naming of the image. Whether successfully solved or not, an “aha!”/no “aha!” decision followed, after which participants rated their feeling during comprehension on a 5-point scale. **b** *Testing phase* The testing phase consisted of an indirect memory test (solving old and new items) and a direct memory test (old/new recognition)

For this purpose, we chose a pictorial problem-solving task in which we presented so-called Mooney stimuli, that is, black and white photos without any shades of gray (Imamoglu, Kahnt, Koch, & Haynes, 2012; Mooney, 1957). The participants’ task during the encoding phase was to try to figure out what was presented in the Mooney image (see Fig. 1a). The encoding phase was incidental, that is, participants were not told that their memory of the Mooney images and their motifs would be tested later on. After 1 week, memory was tested with an indirect (incidental) and a direct (intentional) memory test via solving old and new items and an old/new recognition^3^ memory question, respectively (Richardson-Klavehn & Bjork, 1988). Direct memory tests are more likely to tap into voluntary, controlled retrieval, whereas indirect memory tests are more likely to tap into more automatic, involuntary retrieval (Richardson-Klavehn, 2010). This paradigm enabled us to investigate the insight components generation and “aha!” and their relationships to voluntary (direct test: old/new decision) versus more automatic retrieval processes (indirect test: generating solutions to old items) (see also Schott et al., 2005). We would like to emphasize that we are investigating the relationships of generating a solution and the feeling of “aha!” to long-term memory independently. Thus, we are also testing whether a subjective feeling of “aha!” may also occur when the solution is presented after a failed attempt at generating a solution, and whether this may still be related to learning.

We further incorporated an emotional valence rating of how participants felt when comprehending the solution, in order to compare the quality of the sudden comprehension for generated and non-generated solutions, as well as solutions understood with an “aha!” experience and without.

### Hypotheses

First of all, we hypothesized that both generation and “aha!” would be related to a relatively more positive emotional valence rating than no generation and no “aha!”. In the case of generation accompanied by a feeling of “aha!”, Danek et al. report feelings of joy, tension release, as well as performance-related emotions, such as pride, satisfaction, competitiveness (Danek et al., 2014). While most of these aspects of a positive emotional response can be expected for both generating a solution (with or without “aha!”) or for the “aha!” experience (with or without generation), the performance-related emotions depend on generation and should therefore not be related to the “aha!” experience. Thus, the emotional response is probably slightly more positive for generation than for aha.

In regard to the above discussed characteristics of insight that may be related to learning (Sect. “Insight and long-term memory formation”), we hypothesized that later memory performance should be better for generated solutions during encoding, both for identification and recognition memory performance at test, due to the generation effect. Second, the subjective feeling of “aha!” should lead to enhanced encoding, probably due to the positive emotional response (Danek et al., 2013; Ludmer et al., 2011). Third, as for the relationship between generation and “aha!”, it is unclear what to expect of non-generated solutions, because the relationship between the subjective “aha!” experience and later memory performance has thus far only been tested for generated solutions (Bowden & Jung-Beeman, 2003a; Danek et al., 2013). The implicit assumption of previous studies behind the analysis of aha/no aha responses only for generated solutions was probably that an “aha!” experience can only occur for self-solved items. If so, one might expect an interaction between the variables generation and aha in relation to later memory performance, such that an “aha!” experience is particularly important for later memory of items successfully generated at encoding. However, it could also be that the “aha!” experience can also be invoked by a presented solution after fruitless efforts of trying to solve the problem (Bowden & Jung-Beeman, 2003b), possibly indicating an induced representational change (Ohlsson, 1984, 1992). In this case, the relationships between generation and “aha!” with later memory might be independent.

As for the different measures of memory, we asked (1) whether generation during encoding facilitates identification during testing more than it facilitates recognition memory, which would represent transfer-appropriate processing, and (2) whether the “aha!” experience is more strongly correlated with an increased probability of recognizing the item as old during testing compared to later identification during testing, which might be expected if “aha!” experiences leave behind a distinctive episodic memory trace. The results thus gave an additional opportunity to examine whether successful generation and the “aha!” experience during encoding involve similar or partly distinct processes.

Our data revealed that both the generation of a solution and the “aha!” experience are independently accompanied by a positive emotional response and are also independently related to later memory. As for the different measures of memory, we found that generation at encoding had a stronger relationship to later identification than to later recognition memory at test, whereas the “aha!” experience at encoding had a slightly larger relationship to later recognition memory than to later identification at test.

## Methods

### Participants

Twenty-one healthy young adults participated in the study. One participant had to be excluded, because of perseveration behavior during the aha/no aha decision. Median age of the remaining 20 participants was 23.5 years (range 20–29), 9 participants were female, 11 male. Button assignment to hands was counterbalanced. Participants were included regardless of handedness, because no neural data were acquired (17 were right-handed, one left-handed and two bimanual by self-report). The study was approved by the local ethics committee of the Faculty of Medicine of the Otto-von-Guericke-University. All participants gave written informed consent. They were informed that they had the right to cancel the experiment at any time without any negative personal consequences. Participation was paid with 6 Euros/h.

## Stimulus material

Mooney images were created from 212 square-cut color photos of 137 animate and 75 inanimate objects. The stimuli were chosen from a set of 330 Mooney images that were originally created for the study by Imamoglu et al. (2012). First, a subset of 257 images was selected that contained no more than five exemplars per category and whose motifs were relatively distinct, for example, five different pairs of shoes (high-heels, sneakers, low boots, high boots, sandals). The original color photographs were further labeled and rated for the ambivalence of the motif by four independent raters. Only those images that were rated as non-ambivalent by the majority of raters were included in the final sample of 212 pictures. Images were rated as ambivalent when there was more than one central motif, for example, a man walking with a deer on a street instead of just a man or a deer. The labels of the raters were used to classify the participants’ labels as correct or incorrect. Mooney versions of the original color photos were created by first transforming them to grayscale, smoothing them, and finally setting all shades of gray values below a certain threshold to white, and those above to black (Imamoglu et al., 2012). All of the Mooney images were set to 306 × 306 pixels. Mooney images were presented in the center of a computer screen on a black background in a thin white frame (2 pixels width) to clearly outline the border of the image. Of the 212 images, 159 were randomly chosen for the encoding phase and as old items for the memory test, while the remaining 53 images were used as new stimuli during the memory test.

### Apparatus, task, and procedure

The experiment consisted of two sessions: an incidental encoding phase during which participants tried to identify the central motif of Mooney images, and a testing phase 1 week later that consisted of an indirect and a direct memory test. Participants were not informed that the second session comprised a memory test, but were told that the second session would be relatively similar to the first one with a few changes. This procedure was adopted because incidental encoding has been found to increase the generation effect (Bertsch et al., 2007). Both encoding and testing were done at a standard desktop PC with Windows XP in the same behavioral laboratory. Stimulus presentation and acquisition of behavioral data was controlled with the software Presentation version 16.3 (Neurobehavioral Systems, Berkeley, CA, USA). Stimuli were presented on a 19-in. TFT screen with 60 Hz frame rate and 1280 × 1024 pixels resolution. Responses were made with a standard USB keyboard. During the whole experiment, the experimenter sat in an adjacent room and saw the same display as the participant. Before each session, participants were given written instructions and were asked to verbally summarize the instructions to the experimenter to ensure that they had understood everything as intended. Clarification was provided by the experimenter when needed.

For the encoding phase (see Fig. 1a), participants were asked to try to identify what was presented in each Mooney image within a time limit of 20 s, and to indicate if and when they had done so by pressing the space key. This task is difficult, because the white and black patches of the object cannot be grouped together separately from those of the background. Immediately afterwards they were asked to speak out their answer, for example, “dog”. It was emphasized that participants should only press the button when they were ready to name the motif. When the participant made no response before time ran out, the solution was presented as a grayscale image for 3 s, and they had to name the motif orally. Oral responses were made for both generated and non-generated solutions to ensure that later memory differences were not due to possible additional encoding during spoken identification. As noted above, the correctness was manually scored by the experimenter before data analysis. The solutions that were orally provided by the participants during the experiment were written down during the experiment. This resulted in lists that the experimenter could compare with the labels provided by the raters. After the spoken response, participants were presented with the question “Did you have an ‘aha!’ experience?”, to which they had to press one of two buttons for either “yes” or “no” (left or right arrow key, assignment counterbalanced across participants). The question was presented until participants pressed a button. The written instruction for the encoding phase contained a definition of the subjective “aha!” experience, in an attempt to ensure that the different participants would make relatively comparable decisions about whether they had had an “aha!” experience or not.

After each trial, participants were asked to rate their answer to the question “How did you feel when you recognized the motif?” using a 5-point scale with a range between −2 and 2 (Fig. 1a). Each point on the scale was depicted using smiley figures indicating “very sad”, “sad”, “neutral”, “happy”, and “very happy”. This rating was applicable to both generated and presented solutions. Participants could navigate through the smiley figures (highlighted with a red frame around the smiley) using the left and right arrow keys. The selection had to be confirmed via the space key, which ended the trial. All trials started with a 1-s fixation cross. All stimuli were presented on a black screen. The session consisted of 159 trials, with two short breaks. After the session, participants filled out a questionnaire that contained the items of the novelty seeking scale of the Temperament and Character Inventory (TCI-R, Cloninger, 1999).^4^

After 7 days, at approximately the same hour or, if not possible, at least the same time of day as the encoding phase, participants received an indirect test of their memory of the solutions of the Mooney images (i.e., whether they could identify the object depicted), and their recognition memory of the images themselves, regardless of whether they could identify the item depicted. The initial task in this testing phase was relatively similar to that in the encoding phase (see Fig. 1b). Mooney images were presented for 10 s after the presentation of a fixation cross for 1 s. During that time, participants were instructed to try to identify the item and indicate if they did so by pressing the space button. They were explicitly instructed to ignore whether the image seemed familiar or not, and only to try to identify the item in the image. This was done to ensure that subjects did not consciously try to rely on their recollection of the item and correct solution, as we were interested in incidental memory, i.e., whether one benefits from solving items one has encountered before regardless of whether this is explicitly remembered. Afterwards, they were asked to speak out their answer within 3 s while the question “What is depicted here?” was presented in addition to the Mooney image. If they did not come up with an answer or after they named their solution, the question “Was the picture old or new?” appeared, to which participants were asked to respond via button press (again left and right arrow keys, counterbalanced across participants for “yes” and “no”). Notably, the old/new decision about the Mooney image could be made regardless of whether the participant was able to identify it. However, we cannot know what kind of effect the previous indirect test may have on the direct memory test. This is a very complex relationship. We would like to refer the interested reader to Richardson-Klavehn and Bjork (1988).

At the end of the session, participants filled out a post-experimental questionnaire including the question whether they had used specific strategies to identify the Mooney images, and if so to describe them.

### Design and data analysis

We could not manipulate generation and “aha!” as experimental factors but, at encoding, we expected there to be generated and non-generated solutions as well as solutions identified with and without an “aha!” experience. Therefore, in our main statistical analysis, we analyzed the potential main effects and interaction between the artificial factors GENERATION (generated, non-generated) and AHA (aha, no aha) with repeated-measures analyses of variance (ANOVAs). As dependent variables, we looked at the potential relationships between GENERATION and AHA and the emotional response during the comprehension of the solution in the encoding phase as measured by a 5-point scale, the solution rate of old items in the later indirect identification test, and the hit rate in the direct recognition memory test. The 5-point scale for the emotional response was treated as pseudo-metrical. We further investigated the relationship between AHA and individual-participant median response times (RTs) during generating a solution at encoding, in order to test whether the subjective feeling of “aha!” was dependent on the difficulty of solving an item, for which we used the RTs as an indicator. The median of the emotional response rating and generation RTs per participant was used instead of the mean: (1) because the rating was treated as pseudo-interval-scaled, and (2) because the RTs for each participant had a typical right-skewed distribution so that the median was a better estimate for the peak of the distribution than the mean.

## Results

Data were analyzed with SPSS version 21 (IBM, Armonk, NY) with either related-measures *t* tests or repeated-measures analyses of variance (ANOVAs) where appropriate. Results are reported as significant when *p* < 0.05. As a measure of effect size partial *η* ^2^ is reported for ANOVAs and Cohen’s *d* for *t* tests.

### Dependence between AHA × GENERATION

First, we investigated whether there was a dependency between the “aha!” experience and the generation of a solution. We asked whether participants reported “aha!” experiences uniquely when they recognized the item in the Mooney image without being presented with the solution. We compared the frequency of “aha!” experiences given that the solution was generated [*P*(aha|generated) = 0.47, SD = 0.29] to the frequency of “aha!” experiences given the solution was not generated [*P*(aha|not generated) = 0.59, SD = 0.33] using a related-measures *t* test. There was no significant difference [*t*(19) = 1.45, *p* = 0.173, Cohen’s *d* = 0.386], suggesting that “aha!” experiences did not occur more often for generated compared with non-generated solutions. The descriptive difference even showed a trend for the opposite. Table 1 shows the relative number of cases of all four conditions, e.g., of all Mooney images, 28 % of the motifs were generated with a feeling of “aha!”, 32 % were generated without a feeling of “aha!”, 22 % of the motifs were not generated but the presented solution still induced a feeling of “aha!”, and 18 % were neither generated nor did the presented solution induce a feeling of “aha!”.

**Table 1:** Percentages of Mooney images that were solved (=generated, i.e., the motif has been identified) or not solved and which did or did not produce a subjective “aha!” experience

%	Aha	No aha	Sum
Generated	28 (−2.2)	32 (2.2)	60
Not generated	22 (2.7)	18 (−2.7)	40
Sum	50	50	100

Standard residuals are provided in brackets

Participants did not respond faster or slower to items solved with “aha!” (mean = 4723 ms, SD = 3129 ms) compared to those without “aha!” (mean = 4486 ms, SD = 2059 ms), suggesting that the feeling of “aha!” was not dependent on the difficulty of items, because items more difficult to solve should have led to higher RTs.

### General memory performance

To investigate whether learning occurred during the encoding phase, we first compared the solution rate of old versus new Mooney images at test by means of a related-measures *t* test. Old items (mean = 61 %, SD = 7 %) were significantly more often solved than new items (mean = 45 %, SD = 9 %) [*t*(19) = 11.52, *p* < 0.001, Cohen’s *d* = 1.985]. As for recognition memory performance, the overall hit rate was 63 % (SD = 12 %) and false alarm rate 22 % (SD = 11 %); that is, even after a week the participants’ discrimination was relatively high with 41 % (SD = 11 %).

It should be noted that not all items that were recognized as old were solved again, and vice versa. As can be seen in Table 2, in the testing session on average participants failed to recognize 16 % of the items as old, while they could not solve 18 % of the old Mooney images which they correctly identified as old.

**Table 2:** Distribution of solved old items and correct recognition during the testing phase

%	Solved	Not solved	Sum
Hit	45.7 (6.0)	17.6 (−7.6)	63.3
Miss	16.0 (−7.9)	20.7 (10.0)	36.7
Sum	61.7	38.3	100

Standard residuals are provided in brackets

Table 3 shows the relationship between generation during encoding, solution rates during testing and hit rates. Descriptively, when the solution was not generated during encoding, items had the same likelihood to be correctly recognized as old whether they were solved at testing or not, but when the solution was generated during encoding, the likelihood to be correctly recognized as old was higher when they were also solved at testing. At the same time, the most misses were made if the solution could not be found during encoding and testing. This indicates that the old/new judgment was probably influenced by the preceding attempts during learning and testing at solving an item. However, the dependency between direct and indirect measures of memory such as these is very complex and not completely understood (Richardson-Klavehn & Bjork, 1988). We will therefore refrain from discussing this finding in more detail.

**Table 3:** Distribution of solved old items and correct recognition during the testing phase split for both levels of the variable GENERATION

%	Generated (encoding)		Not generated (encoding)	
	Solved (testing)	Not solved (testing)	Solved (testing)	Not solved (testing)
Hit	59.1	11.7	25.1	26.7
Miss	19.6	9.6	10.5	37.6

### The relationship between GENERATION, AHA, and the emotional response at encoding

**Fig. 2:**
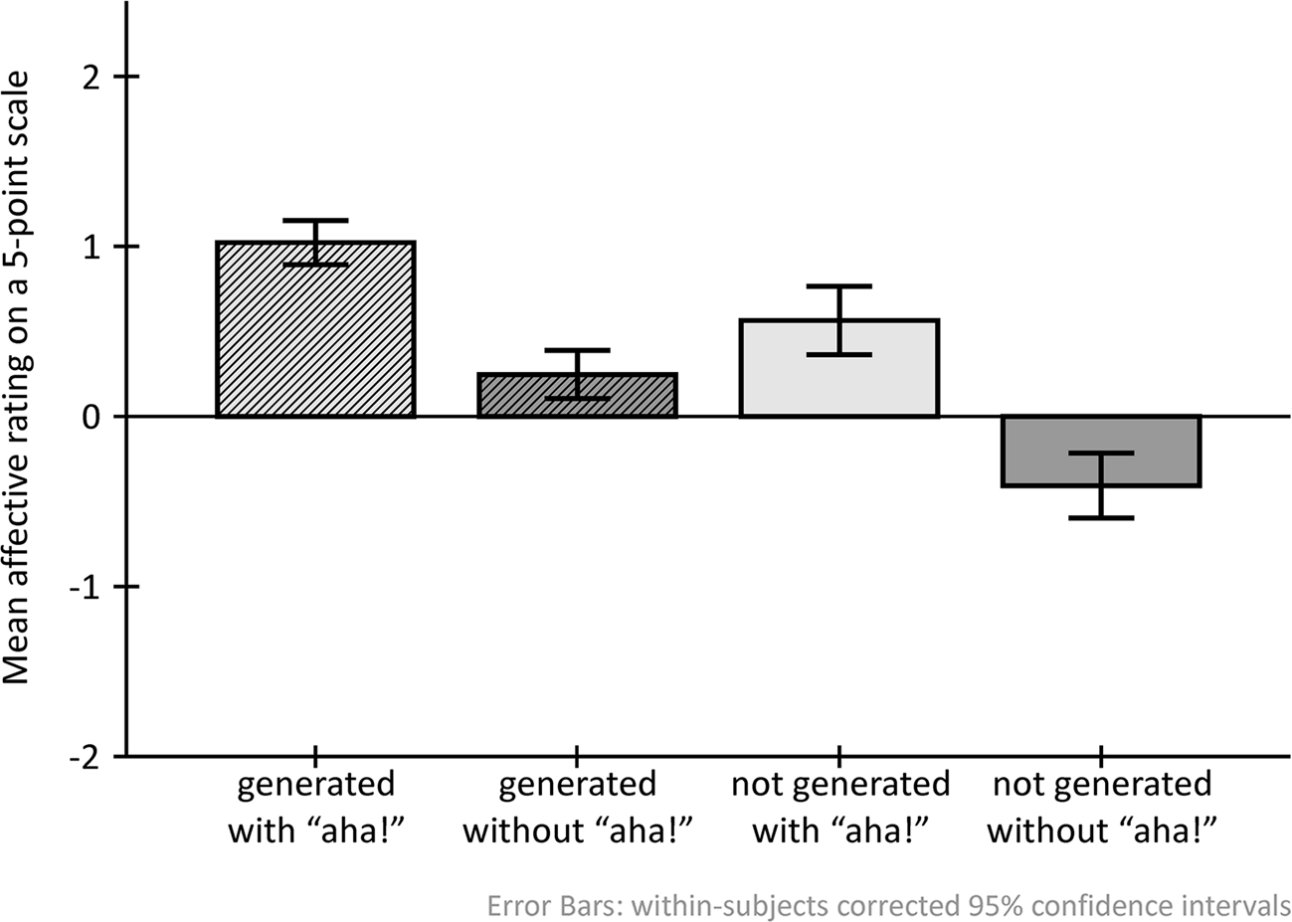
Relationship of GENERATION and AHA with the emotional response. Successful generation and the subjective “aha!” response were independently associated with a relatively more positive rating than no generation and no “aha!”

We were interested in whether the emotional response related to the subjective “aha!”/no “aha!” and generated/non-generated solutions differs qualitatively, and whether the emotional response to either might depend on the other. To analyze this relationship, we conducted a repeated-measures ANOVA with factors GENERATION (generated, non-generated) × AHA(aha, no aha) and the emotional response rating as a dependent variable. Both GENERATION [*F*(1, 19) = 48.6, *p* ≤ 0.001, *η* ^2^ = 0.719] and AHA [*F*(1, 19) = 60.26, *p* ≤ 0.001, *η* ^2^ = 0.760] had significant main effects on the emotional response, whereas there was no interaction, as can be seen in Fig. 2. Generated solutions had an overall mean positive rating of 0.63 (SD = 0.07) while non-generated solutions were almost rated as neutral with a mean of 0.08 (SD = 0.10). Solutions inducing a subjective “aha!” experience were also rated more positively (overall mean = 0.79, SD = 0.09) than those without aha (overall mean = −0.08, SD = 0.10). The absence of an interaction supports the idea that the subjective feeling of “aha!” does not depend on being proud for having solved the item (Topolinski & Reber, 2010).

### The relationship between GENERATION, AHA, and solving old items at testing

**Fig. 3:**
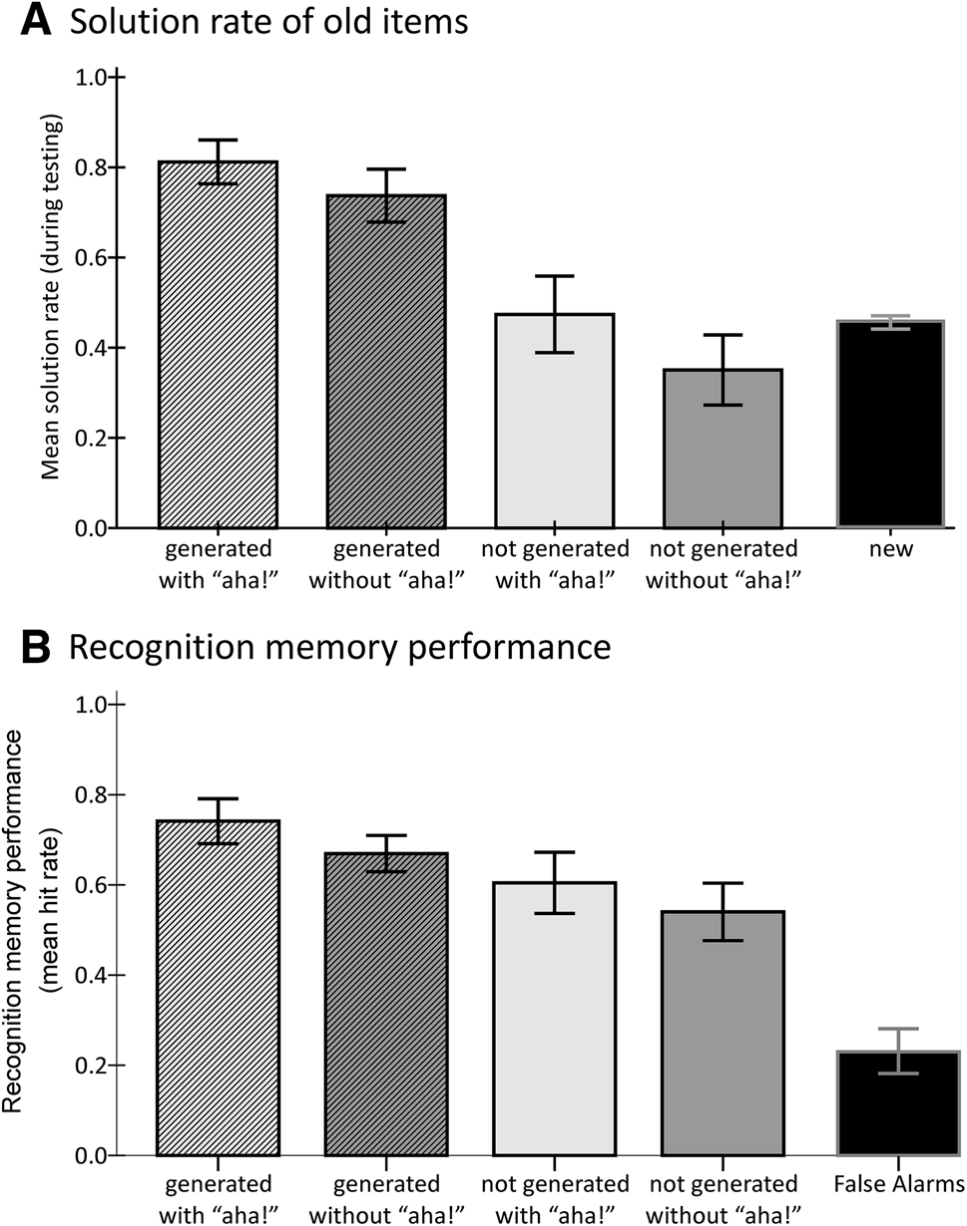
Relationship of GENERATION and AHA to memory performance. **a** Solution rate of new items and old items split for generation and “aha!” during encoding. **b** Recognition memory performance, i.e., false alarm rate and hit rate split for generation and “aha!” during encoding. Generation and “aha!” were associated with higher solution rates of old items and higher hit rates. For both measures of memory these relationships were independent. Error bars represent 95 % confidence intervals, for within-subjects design corrected according to Masson and Loftus (2003)

GENERATION and AHA at encoding showed a similar relationship with our indirect memory performance measure, that is, the solution rate of old items, as they had with the emotional response (see Fig. 3a). The generation of a solution during encoding was associated with a higher solution rate during testing (overall mean = 0.78, SD = 0.02) than when the solution was presented (overall mean = 0.41, SD = 0.03) [*F*(1, 19) = 136.53, *p* ≤ 0.001, *η* ^2^ = 0.878]. Regardless of GENERATION, when the solution was understood with a feeling of “aha!” at encoding, solution rate during testing was higher (overall mean = 0.64, SD = 0.03) than when it was not accompanied by an “aha!” (overall mean = 0.54, SD = 0.04) [*F*(1, 19) = 4.66, *p* = 0.044, *η* ^2^ = 0.197]. As in the previous analysis, there was no interaction. Note that solution rates for old items were only higher than for new items when the solution was generated during encoding. As this might suggest that only easy items were solved repeatedly, we tested the dependency between item difficulty and learning (see Sect. “Item difficulty, learning, and “aha!””).

### The relationship between GENERATION, AHA, and recognition memory performance

As can be seen in Fig. 3b, GENERATION and AHA also had independent relationships with the hit rate during the recognition memory test, with generated solutions and non-generated (presented) solutions that induced an “aha!” experience being associated with a higher later recognition rate. The main effect of GENERATION was significant with *F*(1, 19) = 16.50, *p* = 0.001, *η* ^2^ = 0.465 (see Fig. 3b). Items whose solution could be generated during encoding were significantly more often correctly recognized as old (overall mean = 0.71, SD = 0.03) than items whose solution was presented (overall mean = 0.57, SD = 0.04). The relationship between GENERATION and recognition memory performance was weaker than that of GENERATION and later solution rates during testing (cf. Fig. 3a). As for the AHA factor, items that induced an “aha!” experience during encoding were associated with a significantly higher recognition rate (overall mean = 0.67, SD = 0.03) than those which did not induce an “aha!” (overall mean = 0.61, SD = 0.04) [*F*(1, 19) = 5.95, *p* = 0.025, *η* ^2^ = 0.238]. As with the emotional response and solution rates during testing, there was no interaction.

All in all, the pattern of relationships between the factors GENERATION, AHA, and the measures of the emotional response, solution rate of old items, and recognition memory performance was very consistent.

### Comparison between both measures of memory, GENERATION, and AHA

To test our hypotheses that generation may have a stronger relationship to solving old items at test, while the “aha!” experience might have a stronger relationship to recognizing old items, we computed a 2 × 2 repeated-measures ANOVA with factors TEST (solving old items, recognition memory) and INSIGHT_ASPECT (generation, aha). As a dependent variable, we compared the differences between generated minus non-generated memory performances (generation effect) and aha minus no aha memory performances (aha effect) in both our direct and indirect measures of memory. The ANOVA revealed a main effect of TEST [*F*(1, 19) = 22.20, *p* < 0.001, *η* ^2^ = 0.539], a main effect of INSIGHT_ASPECT [*F*(1, 19) = 13.55, *p* = 0.002, *η* ^2^ = 0.416], and an interaction [*F*(1, 19) = 14.09, *p* = 0.001, *η* ^2^ = 0.426]. To resolve the interaction, we compared the generation effect and the aha effect for solving old items vs. recognizing old items during test separately with related-measures *t* tests. The *t* test for the generation effect revealed a significantly higher generation effect for solving old items (mean = 0.36, SD = 0.14) in comparison to recognizing old items (mean = 0.13, SD = 0.15) during test [*t*(19) = 7.27, *p* < 0.001, Cohen’s *d* = 1.585]. As for the aha effect, we found no significant difference between solving old items (mean = 0.10, SD = 0.21) and recognizing old items (mean = 0.07, SD = 0.12) during test [*t*(19) < 1, *p* > 0.4, Cohen’s *d* = 0.175]. We further tested whether the generation effect or the aha effect was larger in solving old items and recognizing old items during test. Here we found that the generation effect was significantly higher than the aha effect in solving old items during testing [*t*(19) = 4.40, *p* < 0.001, Cohen’s *d* = 1.457], whereas—although descriptively double the size—only showing a trend towards significance in recognizing old items [*t*(19) = 1.54, *p* = 0.141, Cohen’s *d* = 0.442].

To summarize, generation had a considerably stronger relationship than the subjective “aha!” experience to solving old items during testing. Moreover, generation had a stronger relationship with solving old items during testing than with recognition memory performance, while the relationship of the “aha!” experience to later memory was comparable in both tests.

### Item difficulty, learning, and “aha!”

**Fig. 4:**
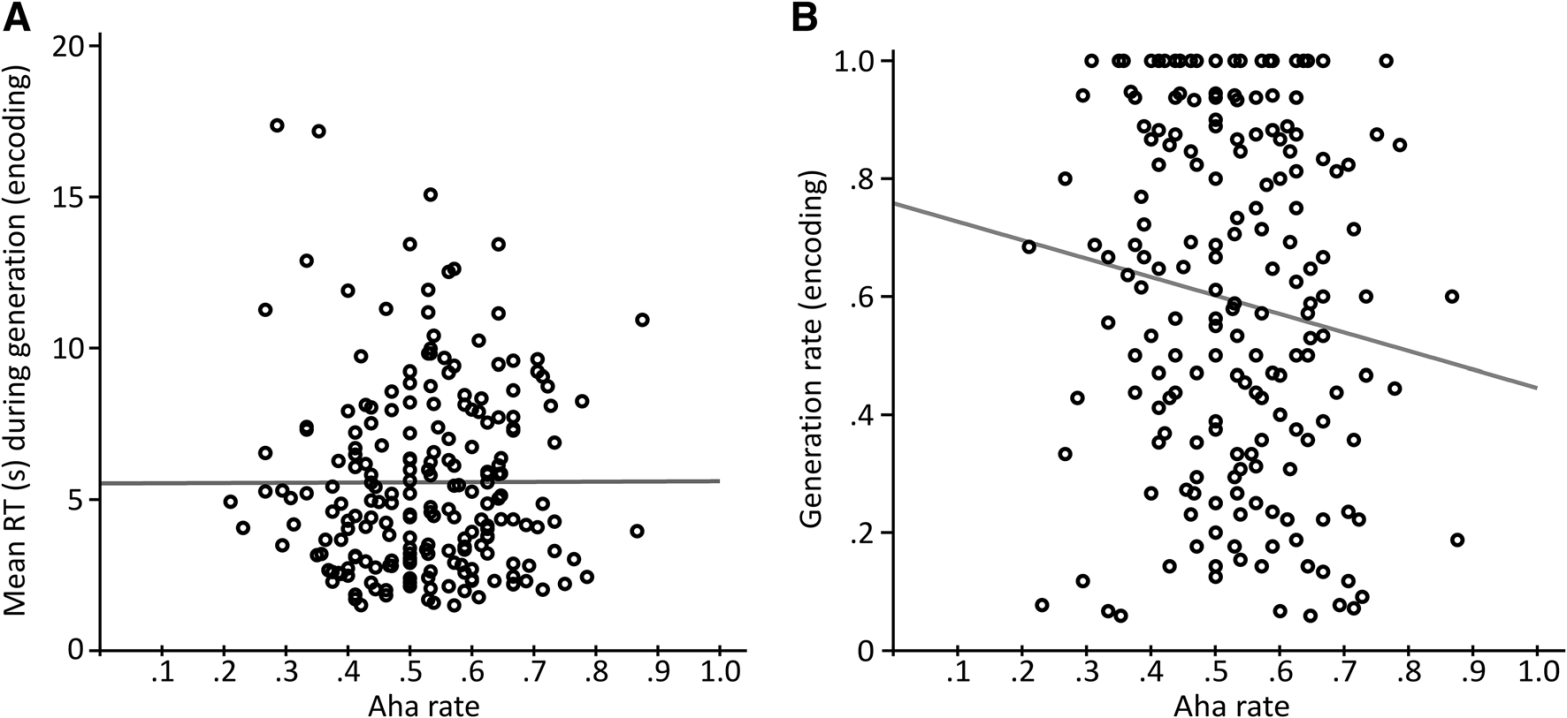
Relationship between item difficulty and “aha!” frequency. **a** RT for generated items × aha rate. The rate of “aha!” responses did not correlate with RTs for generated solutions. **b** Generation rate × aha rate. The “aha!” rate did correlate negatively with the generation rate during testing (*r* = −0.128). However, considerable ceiling effects, as visible in **b**, show that the relationship should be interpreted with caution

To investigate whether difficult items, that is, items that were solved with higher RTs or items solved less frequently, induced “aha!” experiences more often, we aggregated the data split for the 212 items across the 20 participants. One-tailed Pearson correlation analyses were executed to analyze the relationship between median RTs and “aha!” frequency across items (i.e., median RT across subjects per item, relative number of participants who rated “aha!” per item) as well as between the frequency of generated solutions and frequency of “aha!” experiences. As can be seen in Fig. 4a, there was no correlation between median RT and percent “aha!” [*N* = 210 due to 2 items that were solved by none of the participants, *r* = 0.003]. There was a moderate negative correlation between the generation rate and the “aha!” rate during encoding [*N* = 212, *r* = −0.128, *p* = 0.031], that is, higher “aha!” rates correlated with lower generation rates. This result should, however, be interpreted with caution due to considerable ceiling effects: some items could be solved by all participants as can be seen in Fig. 4b.

**Fig. 5:**
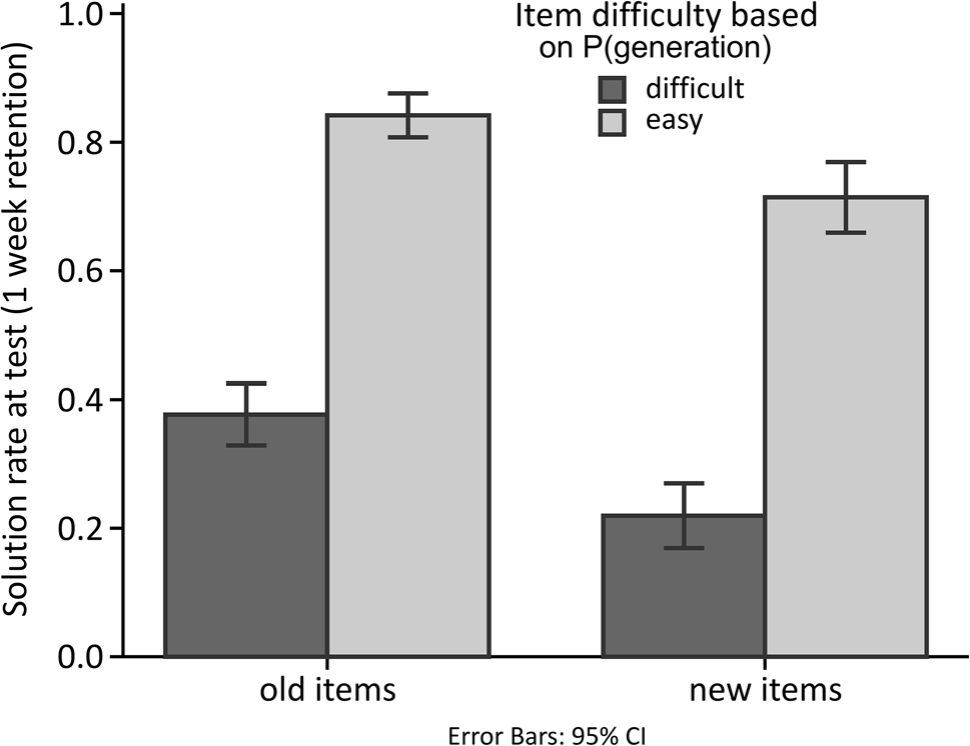
Learning effect for easy and difficult items as measured by the generation rate at test for old minus new items. There was no significant difference in the size of the learning effect for difficult compared to easy items

As it could potentially have been the case that the learning effect in the indirect test described previously is only due the repeated generation of easy items, and thus not real learning, we split items into difficult and easy items based on the median of the generation rate (median = 0.60), that is, how many participants could solve an item when it was presented during encoding. As learning rate we subtracted the generation rate of items during test when they were new from when they were old, and compared the learning rate for difficult and easy items (see Fig. 5). Both categories showed a significant learning effect of 0.16 [SD = 0.21; *t*(104) = 7.84, *p* < 0.001] for difficult and 0.13 [SD = 0.23; *t*(106) = 5.68, *p* < 0.001] for easy items when tested with a one-sample *t* test against 0. Importantly, there was no significant difference between the learning rate for difficult and easy items [independent-sample *t*(210) = 0.984, *p* = 0.326, Cohen’s *d* = 0.136], corroborating that the generation during encoding had the same beneficial learning effect for difficult and easy items. Descriptively, learning was even higher for difficult items.

## Discussion

In this study, we used a new paradigm that enabled us to investigate generation, that is, self-solved vs. presented solutions, and the subjective “aha!” experience in regard to later LTM performance, to gain a better understanding of the different components of insight that may have a beneficial effect on encoding. For this purpose, we used a pictorial insight paradigm in which participants were asked to identify motifs of Mooney images and to rate their subjective “aha!” experiences (Imamoglu et al., 2012; Mooney, 1957).

### Generation and “Aha!” are independently related to later memory performance

Our findings support the idea that “aha!” experiences as well as the successful generation of an insight solution are accompanied by a positive feeling (Fig. 2). In addition, the emotional response towards the feeling of “aha!” was independent of the emotional response towards the generation of the solution. Thus, although generation with “aha!” was accompanied by the most positive feeling, the positive feeling was not dependent on the joint occurrence of both; the effect is additive. In line with Sternberg’s suggestion of interpreting an additive effect as reflecting different (stages of) cognitive processes (Sternberg, 1969), this result may indicate that different aspects that contribute to the emotional valence rating are associated with the generation of a solution and the subjective “aha!” experience. This interpretation would also be in line with Topolinski and Reber who propose that the subjective “aha!” experience reflects something other than pride, as which one may interpret the positive emotional response related to generation (Topolinski & Reber, 2010).

We hypothesized that both generation and “aha!” would enhance LTM encoding on the one hand due to the rewarding, positive emotional response that accompanies novel understanding or successful generation, and on the other hand, because the generation of a solution leads to a better integration of the information into memory, possibly due to high schema-consistency. Furthermore, the “aha!” experience may reflect that the novel perception of the Mooney as an object instead of a random composition of black and white blotches came suddenly and was especially strong, clear, and convincing, thereby leaving a stronger memory trace (Ludmer et al., 2011). According to previous studies that looked only at generated solutions that were either accompanied by an “aha!” or not, but not at non-generated solutions (e.g., Bowden & Jung-Beeman, 2003a; Danek et al., 2013), one could have expected that the subjective “aha!” experience only affects behavior when solutions were generated. Whether this is actually the case has not been tested in any studies the authors are aware of. Here, our data suggest that this is not the case. Not only did generation and “aha!” have independent relationships to the emotional response, we also found that both generation and the subjective “aha!” experience were independently related to memory performance in our indirect memory test of solving old items, as well as in our direct old/new recognition memory test. Thus, “aha!” and generation seem to be independently related to later memory besides being independently associated with emotional valence.

Regarding our main question at hand, that is, which components of insight contribute in which way to a beneficial effect on LTM encoding, our results suggest that it is the generation effect which has the strongest relation to later memory performance, whereas the relationship between “aha!” and later memory is relatively weak (Fig. 3). A previous study had already shown that generation accompanied by the subjective “aha!” is associated with better later memory performance than generation without “aha!” (Danek et al., 2013). Danek et al. found that memory for solutions of magic tricks was enhanced for those solutions which were generated with “aha!” compared to those generated without “aha!” in the learning phase. In the current study, because generation and “aha!” had additive relationships with later memory, generation accompanied by an “aha!” experience indeed showed the best memory performance, but not in the interactive way one might expect. Our findings do corroborate the relationship of the subjective “aha!” with enhanced memory for the solution. In addition, our study shows that the subjective “aha!” may even occur when the solution is presented after a failed attempt at generation and that this can also enhance later memory for the solution.

Regarding our hypotheses that generation may have a stronger relationship with our indirect memory test (solving old items during testing), which measured more automatic retrieval processes, while “aha!” may have a stronger relationship with our direct memory test (recognition memory performance), which measured more voluntary, conscious retrieval, than with our indirect memory test, we found support for the first, but none for the latter. Given that our indirect memory test was essentially the same as our encoding task, transfer-appropriate processing would easily explain the difference between the generation effect on solving old items vs. correctly recognizing old items (Morris et al., 1977). As Bowers already suggested, during the search process for a solution, spreading activation may activate semantic memory content potentially relevant for the solution (Bowers et al., 1990; Collins & Loftus, 1975). If generation fails, it may be due to the automatically activated information leading to an impasse that could not be overcome. If the testing situation then activates the neural pattern similar to the one at encoding, this is only beneficial for successful generation, but not necessarily for failed generation. However, transfer-appropriate processing may not explain the generation effect per se, as discussed in the Introduction. Moreover, the recognition memory task always followed the generation task during testing, hence it may well be that the previous engagement with the items enhanced recognition memory performance more for those items for which participants had already failed to generate the solution during encoding. This is also supported by the higher hit rate for old items that were not solved during testing and whose solution was also not generated during encoding compared to those items for which generation during encoding was successful (see Table 3).

Taken together, it seems as though the subjective “aha!” experience was only an additive component to the larger generation effect, both in regard to later memory and the emotional response. The “aha!” experience itself has a comparably weak relationship to later memory performance. The finding that its relationship to later memory is statistically comparable for solving old items during testing and recognizing old items may suggest that it influences recollection, which is thought to reflect the retrieval of qualitative information about an episode more than familiarity, a more global measure of memory strength (Yonelinas, Otten, Shaw, & Rugg, 2005; Yonelinas, Aly, Wang, & Koen, 2010). This interpretation is also supported by the emotional response.

These findings suggest that it is important that learners try to solve problems on their own, because even if they are not able to come up with the solution themselves, when the revealed solution is highly surprising and compelling, this might still enhance learning. In regard to education, our data would support the importance of problem-based learning (Loyens, Kirschner, & Paas, 2012), that is, the importance of learners to engage in a creative problem-solving process.

### Overlaps and differences with previous studies

How do our results further our understanding of learning from insight? Previous studies on insight and learning have shown that generating solutions to problems that do not readily come to mind, but which warrant an “effort towards understanding”, i.e., comprehension which is preceded by a state of incomprehension, enhance later memory for the solution (Auble et al., 1979; Mednick, 1962; Wills, Soraci, Chechile, & Taylor, 2000). For example, by comparing sentences that are not immediately comprehensible without a cue with sentences that are comprehensible, because the cue word is integrated into the sentence (Auble et al., 1979), Auble and colleagues realized a paradigm that is highly identical with the typical generation effect manipulation, that is, generate versus read conditions (Slamecka & Graf, 1978).^5^ It is difficult to say whether the generation effect for insight problems is special. It has been proposed that this specific type of generation effect is different from the well-known generation effect of the main corpus of studies on this effect reported in the long-term memory literature (Wills et al., 2000). Wills and colleagues reasoned that the typical generation studies try to make the generation condition very easy to reach a 100 % solution rate and to avoid item selection effects, that is only easy items being solved (e.g., Gardiner, Java, & Richardson-Klavehn, 1996). However, from this one could infer that the generation effect for “insight” problems is just a generation effect for relatively difficult problems. On the other hand, it could be that the particular nature of insight problem-solving benefits learning even more than analytical problem solving.

Support for the notion that not only generation per se, but generating the solution in a very specific way, namely accompanied by a feeling of “aha!”, comes from Danek et al. (2013). Based on self-reports of the participants, they distinguished between items solved with an “aha!” experience—a sudden realization of the solution of which one is very confident—and more analytically—a gradual and stepwise discovery of the solution of which one is less confident. Thus, in contrast to Bowden and Jung-Beeman (2003a) they did not just assume that the no “aha!” response reflects analytical problem-solving, but specifically defined it in this way for the participants. They found that solving problems with “aha!” led to better memory encoding than solving without “aha!”. This result suggests that learning from insight is not just supported by the generation effect, but by a very specific solution path that is mostly unconscious (Reber, Ruch-Monachon, & Perrig, 2007). However, our data suggest that this solution must not necessarily be found by the participants themselves. It seems that it suffices that participants attempt to solve a problem, followed by experiencing the revealed solution and sudden comprehension with an “aha!” experience, as described previously.

### Theoretical considerations on learning from insight

By what means can both the generation of a solution and the experience of an “aha!”, i.e., suddenness of understanding and being convinced of the truth of the solution, enhance learning? In the Introduction, besides the potential explanations for the classical generation effect, we considered the following mechanisms: novelty, intrinsic reward, and schema-consistency. Our results support the idea that intrinsic reward and novelty are one of the reasons that lead to better memory, since both generation and the “aha!” experience are, in accordance with the novelty exploration bonus hypothesis (Kakade & Dayan, 2002), associated with a relatively more positive emotional response and better memory. Evidence suggests that reward enhances hippocampal encoding by means of dopaminergic projections from the midbrain (Kumaran & Maguire, 2009; Lisman & Grace, 2005). Our results support the idea that insight enhances learning partially by means of being an intrinsic reward.

Generating the solution indicates that the visual information of the Mooney image has been mentally reorganized (Mayer, 1995). Evidence even suggests that a literal representational change occurred: the neural representation of the Mooney image now resembles that of the grayscale image more than that of the Mooney image before comprehension (Hsieh, Vul, & Kanwisher, 2010). Another study by Imamoglu et al. suggests that feed-forward input from the prefrontal cortex influences object recognition after the motif of the Mooney image has been identified (Imamoglu et al., 2012). Both findings can be interpreted as evidence for the novel information (Mooney image) being associated with semantic knowledge which changes the way the Mooney image is perceived from mere black and white blotches to a coherent figure. Generating the solution may therefore enhance encoding by means of detecting schema-consistency which has been reported to accelerate or even shortcut hippocampal encoding (Tse et al., 2011; Van Kesteren et al., 2013).

Schema-consistency may not only be detected during successful generation, but may also be indicated when participants report an “aha!” experience for presented solutions after failed generation. However, they did not reach the representational change that led to the detection of schema-consistency themselves which could explain why generation is superior to the “aha!” experience in regard to later memory performance. It is conceivable that the “aha!” experience for non-generated solutions occurs mainly for items whose solution was close to surfacing into consciousness, similar to the “incomplete generation” explanation of Slamecka and Fevreiski (1983) (they reported that later memory for the solution shows the pattern generate ≤ fail-to-generate < read, whereby the read and fail-to-generate condition differ in that subjects did not have the chance to solve the item in the read condition, but were presented with the solution immediately). In line with Bowers’ two-stage model of intuition (Bowers et al., 1990), the first stage of forming an intuitive feeling of coherence may have been reached, but not the surfacing of a hypothesis about the solution into consciousness (=insight). The last step would then be induced by the presentation of the solution after the time for solving the item ran out. Unfortunately, we have no index of the participants’ closeness to the solution to evaluate the hypothesis that the “aha!” experience in failed generation reflects an incomplete representational change that is accelerated by the presented solution. We are currently conducting a study on the relationship between generation, “aha!”, and the feeling-of-warmth to later memory to illuminate this matter.

## Conclusion

The current study revealed that successful generation and the subjective feeling of “aha!” were independently beneficially related to LTM encoding as assessed by two different types of memory tests. This is in contrast to the hypothesis that “aha!” experiences would especially be beneficially related to LTM formation when accompanying successful generation—as might be expected intuitively and based on prior literature. The results suggest, by contrast, that a sudden revelation of the solution that induces a comparable feeling to the “aha!” for self-solved items can also be beneficial for LTM encoding, even though encoding lacks the benefit of generation itself. This could possibly be due to the “aha!” experience for presented solutions indicating incomplete representational change. Studies investigating insight problem-solving, and especially the few of them which assessed the impact of insight on LTM encoding, have so far failed to take into account both factors at the same time in a fully crossed manner, thereby overlooking the possible effect the “aha!” experience can have even when the solution is presented after failed attempts to solve an insight problem. We suggest that future studies should always take both factors into account. The result that generation and the subjective “aha!” experience are independently related to later memory may further suggest that they affect the encoding process on different processing levels (Richardson-Klavehn, 2010; Sternberg, 1969). Electrophysiological and neuroimaging studies could therefore tackle this hypothesis by investigating potential differences in neural correlates of those processes.

## Footnotes

1 It has to be noted that generation and the subjective “aha!” experience are not the only criteria that have been used to operationalize insight. However, these are two criteria that may be especially relevant to learning from insight and which we therefore chose for further investigation. This was done without a specific assumption of how these aspects contribute to the definition of insight. Our interest was in how generation and the subjective “aha!” experience contribute to learning, not in their importance for the definition of insight.

2 It should be noted that Topolinski and Reber distinguish between the nature of the positive feeling related to the subjective “aha!” and generation of a solution; they explicitly state that the emotional response begins before the assessment of the solution and that it therefore cannot be pride.

3 To avoid misunderstandings, the term *recognition* is solely used to refer to recognition memory, while we refer to recognizing the motif of a Mooney, as in object recognition, only as *identifying* a Mooney image.

4 The TCI’s novelty seeking scale was administered based on the idea that high novelty seeking scores may have a positive relationship with insight problem-solving and learning from insight. In a study by Krebs et al. (2009) it was reported that novelty-seekers were more responsive (in terms of the BOLD signal) to novel cues in the absence of reward and needed less reward to boost their memory for novel cues. We therefore hypothesized that novelty-seekers would show better memory performance. However, no such correlation was found.

5 It should be noted that the generation effect literature makes a distinction between a read condition in which one does not have the chance to generate in contrast to a fail-to-generate condition. Both also have different effects on later memory, with fail-to-generate scoring between read and generation, although fail-to-generate and read are both less beneficial to memory than successful generation.

## References

[CR1] Anderson JR (1983). A spreading activation theory of memory. Journal of Verbal Learning and Verbal Behavior.

[CR2] Auble P, Franks J, Soraci S (1979). Effort toward comprehension: Elaboration or “aha”?. Memory & Cognition.

[CR3] Bertsch S, Pesta BJ, Wiscott R, McDaniel MA (2007). The generation effect: A meta-analytic review. Memory & Cognition.

[CR4] Bowden EM, Jung-Beeman M (2003). Normative data for 144 compound remote associate problems. Behavior Research Methods, Instruments, & Computers.

[CR5] Bowden EM, Jung-Beeman M (2003). Aha! Insight experience correlates with solution activation in the right hemisphere. Psychonomic Bulletin & Review.

[CR6] Bowden EM, Jung-Beeman M, Fleck J, Kounios J (2005). New approaches to demystifying insight. Trends in Cognitive Sciences.

[CR7] Bowers KS, Regehr G, Balthazard C, Parker K (1990). Intuition in the context of discovery. Cognitive Psychology.

[CR8] Bühler, K. (1907). Tatsachen und Probleme zu einer Psychologie der Denkvorgänge. I: Über Gedanken. In *Archiv für die Gesamte Psychologie 9.*

[CR9] Bunzeck N, Doeller CF, Dolan RJ, Düzel E (2012). Contextual interaction between novelty and reward processing within the mesolimbic system. Human Brain Mapping.

[CR10] Burns DJ (1992). The consequences of generation. Journal of Memory and Language.

[CR11] Cloninger CR (1999). The temperament and character inventory–revised.

[CR12] Collins AM, Loftus EF (1975). A spreading-activation theory of semantic processing. Psychological Review.

[CR13] Danek AH, Fraps T, von Müller A, Grothe B, Ollinger M (2013). Aha! experiences leave a mark: Facilitated recall of insight solutions. Psychological Research.

[CR14] Danek AH, Fraps T, von Müller A, Grothe B, Öllinger M (2014). It’s a kind of magic—what self-reports can reveal about the phenomenology of insight problem solving. Frontiers in Psychology.

[CR15] Gardiner JM, Java RI, Richardson-Klavehn A (1996). How level of processing really influences awareness in recognition memory. Canadian Journal of Experimental Psychology/Revue canadienne de psychologie expérimentale.

[CR16] Hsieh P-J, Vul E, Kanwisher N (2010). Recognition alters the spatial pattern of FMRI activation in early retinotopic cortex. Journal of Neurophysiology.

[CR17] Imamoglu F, Kahnt T, Koch C, Haynes J-D (2012). Changes in functional connectivity support conscious object recognition. NeuroImage.

[CR18] Jung-Beeman M, Bowden EM, Haberman J, Frymiare JL, Arambel-Liu S, Greenblatt R (2004). Neural activity when people solve verbal problems with insight. PLoS Biology.

[CR19] Kakade S, Dayan P (2002). Dopamine: Generalization and bonuses. Neural Networks.

[CR20] Klein G, Jarosz A (2011). A naturalistic study of insight. Journal of Cognitive Engineering and Decision Making.

[CR21] Kounios J, Beeman M (2009). The Aha! moment: The cognitive neuroscience of insight. Current Directions in Psychological Science.

[CR22] Krebs RM, Schott BH, Düzel E (2009). Personality traits are differentially associated with patterns of reward and novelty processing in the human substantia nigra/ventral tegmental area. Biological Psychiatry.

[CR23] Kumaran D, Maguire EA (2009). Novelty signals: A window into hippocampal information processing. Trends in Cognitive Sciences.

[CR24] Lisman JE, Grace AA (2005). The hippocampal-VTA loop: Controlling the entry of information into long-term memory. Neuron.

[CR25] Loyens, S. M. M., Kirschner, P. A., & Paas, F. (2012). Problem-based learning. In K. R. Harris, S. Graham, T. Urdan, A. G. Bus, S. Major, & H. L. Swanson (Eds.), *APA Educational Psychology Handbook: Vol. 3. Application to learning and teaching* (Vol. 2). Washington: American Psychological Association. doi:10.1037/13275-000.

[CR26] Ludmer R, Dudai Y, Rubin N (2011). Uncovering camouflage: Amygdala activation predicts long-term memory. Neuron.

[CR27] Masson MEJ, Loftus GR (2003). Using confidence intervals for graphically based data interpretation. Canadian Journal of Experimental Psychology/Revue Canadienne de Psychologie Expérimentale.

[CR28] Mayer RE, Sternberg RJ, Davidson JE (1995). The search for insight: Grappling with gestalt psychology’s unanswered questions. The nature of insight.

[CR29] McElroy LA, Slamecka NJ (1982). Memorial consequences of generating nonwords: Implications for semantic-memory interpretations of the generation effect. Journal of Verbal Learning and Verbal Behavior.

[CR30] McNamara DS, Healy AF (2000). A procedural explanation of the generation effect for simple and difficult multiplication problems and answers. Journal of Memory and Language.

[CR31] Mednick SA (1962). The associative basis of the creative process. Psychological Review.

[CR32] Mooney CM (1957). Age in the development of closure ability in children. Canadian Journal of Psychology/Revue canadienne de psychologie.

[CR33] Morris CD, Bransford JD, Franks JJ (1977). Levels of processing versus transfer appropriate processing. Journal of Verbal Learning and Verbal Behavior.

[CR34] Ohlsson S (1984). Restructuring revisited I. Summary and critique of the Gestalt theory of problem solving. Scandinavian Journal of Psychology.

[CR35] Ohlsson S, Keane M, Gilhooley K (1992). Information-processing explanations of insight and related phenomena. Advances in the psychology of thinking.

[CR36] Öllinger M, Jones G, Knoblich G (2013). The dynamics of search, impasse, and representational change provide a coherent explanation of difficulty in the nine-dot problem. Psychological Research.

[CR37] Otmakhova, N., Duzel, E., Deutch, A. Y., & Lisman, J. (2013). The hippocampal-VTA loop: the role of novelty and motivation in controlling the entry of information into long-term memory. In G. Baldassarre & M. Mirolli (Eds.), *Intrinsically Motivated Learning in Natural and Artificial Systems* (pp. 235–254). Berlin, Heidelberg: Springer Berlin Heidelberg. doi:10.1007/978-3-642-32375-1.

[CR38] Reber R, Ruch-Monachon MA, Perrig WJ (2007). Decomposing intuitive components in a conceptual problem solving task. Consciousness and Cognition.

[CR39] Richardson-Klavehn A, Mace JH (2010). Priming, automatic recollection, and control of retrieval toward an integrative retrieval architecture. The act of remembering: Toward an understanding of how we recall the past.

[CR40] Richardson-Klavehn A, Bjork RA (1988). Measures of memory. Annual Review of Psychology.

[CR41] Schott BH, Henson RN, Richardson-Klavehn A, Becker C, Thoma V, Heinze H-J, Düzel E (2005). Redefining implicit and explicit memory: The functional neuroanatomy of priming, remembering, and control of retrieval. Proceedings of the National Academy of Sciences of the United States of America.

[CR42] Slamecka NJ, Fevreiski J (1983). The generation effect when generation fails. Journal of Verbal Learning and Verbal Behavior.

[CR43] Slamecka NJ, Graf P (1978). The generation effect: Delineation of a phenomenon. Journal of Experimental Psychology: Human Learning & Memory.

[CR44] Sternberg S (1969). The discovery of processing stages: Extensions of Donders’ method. Acta Psychologica.

[CR45] Topolinski S, Reber R (2010). Gaining insight into the “aha” experience. Current Directions in Psychological Science.

[CR46] Tse, D., Langston, R. F., Kakeyama, M., Bethus, I., Spooner, P. A, Wood, E. R., et al. (2007). Schemas and memory consolidation. *Science*, *316*(5821), 76–82. doi:10.1126/science.1135935. 10.1126/science.1135935 17412951

[CR47] Tse D, Takeuchi T, Kakeyama M, Kajii Y, Okuno H, Tohyama C (2011). Schema-dependent gene activation and memory encoding in neocortex. Science.

[CR48] Van Kesteren MTR, Beul SF, Takashima A, Henson RN, Ruiter DJ, Fernández G (2013). Differential roles for medial prefrontal and medial temporal cortices in schema-dependent encoding: From congruent to incongruent. Neuropsychologia.

[CR49] Van Kesteren MTR, Ruiter DJ, Fernández G, Henson RN (2012). How schema and novelty augment memory formation. Trends in Neurosciences.

[CR50] Vollmers, B. (2014). Intentionalität und Perspektivität—Überlegungen zu einer integrativen kognitiv-phänomenologischen Lerntheorie aus Subjektperspektive. *bwp@ Berufs- und Wirtschaftspädagogik—online*, *26*, 1–13.

[CR51] Wills TW, Estow S, Soraci SA, Garcia J (2006). The aha effect in groups and other dynamic learning contexts. The Journal of General Psychology.

[CR52] Wills TW, Soraci SA, Chechile RA, Taylor HA (2000). “Aha” effects in the generation of pictures. Memory & Cognition.

[CR53] Yonelinas AP, Aly M, Wang W-C, Koen JD (2010). Recollection and familiarity: Examining controversial assumptions and new directions. Hippocampus.

[CR54] Yonelinas AP, Otten LJ, Shaw KN, Rugg MD (2005). Separating the brain regions involved in recollection and familiarity in recognition memory. The Journal of neuroscience: The official journal of the Society for Neuroscience.

